# Functional roles of FAP-α in metabolism, migration and invasion of human cancer cells

**DOI:** 10.3389/fonc.2023.1068405

**Published:** 2023-03-01

**Authors:** Noriko Mori, Jiefu Jin, Balaji Krishnamachary, Yelena Mironchik, Flonné Wildes, Farhad Vesuna, James D. Barnett, Zaver M. Bhujwalla

**Affiliations:** ^1^ Division of Cancer Imaging Research, The Russell H. Morgan Department of Radiology and Radiological Science, The Johns Hopkins University School of Medicine, Baltimore, MD, United States; ^2^ Sidney Kimmel Comprehensive Cancer Center, The Johns Hopkins University School of Medicine, Baltimore, MD, United States; ^3^ Department of Radiation Oncology and Molecular Radiation Sciences, The Johns Hopkins University School of Medicine, Baltimore, MD, United States

**Keywords:** FAP-α, metabolism, migration, invasion, breast cancer, magnetic resonance spectroscopy, wound healing

## Abstract

Fibroblast activation protein-α (FAP-α) is a transmembrane serine protease that is attracting significant interest as it is expressed by a subgroup of cancer-associated fibroblasts that play a role in immune suppression and cancer metastasis. FAP-α is also expressed by some cancer cells, such as melanoma, colorectal and breast cancer cells. Triple negative breast cancer (TNBC) is an aggressive cancer that urgently requires identification of novel targets for therapy. To expand our understanding of the functional roles of FAP-α in TNBC we engineered a human TNBC cell line, MDA-MB-231, to stably overexpress FAP-α and characterized changes in metabolism by ^1^H magnetic resonance spectroscopy, cell proliferation, migration characterized by wound healing, and invasion. FAP-α overexpression resulted in significant alterations in myoinositol, choline metabolites, creatine, and taurine, as well as a significant increase of migration and invasion, although proliferation remained unaltered. The increase of migration and invasion are consistent with the known activities of FAP-α as an exopeptidase and endopeptidase/gelatinase/collagenase in tissue remodeling and repair, and in cell migration. We additionally determined the effects of FAP-α overexpression on the human fibrosarcoma HT1080 cell line that showed increased migration, accompanied by limited changes in metabolism that identified the dependency of the metabolic changes on cell type. These metabolic data identify a previously unknown role of FAP-α in modifying cancer cell metabolism in the TNBC cell line studied here that may provide new insights into its functional roles in cancer progression.

## Introduction

Fibroblast activation protein α (FAP-α) is a dimeric 170-kDa membrane-bound protease that has exopeptidase and endopeptidase/gelatinase/collagenase activity (1–4). FAP-α is overexpressed in more than 90% of reactive stromal fibroblasts of human epithelial cancers, including colorectal, bladder, breast, ovarian, and lung carcinomas (5, 6). FAP-α-positive cancer associated fibroblasts (CAFs) have been found to induce immunosuppression *via* STAT3-CCL2 signaling (7), and by promoting immune checkpoint blockade resistance (8). Although primarily expressed by CAFs, FAP-α expression has also been detected in breast cancer, astrocytoma, melanoma, colorectal cancer, and oral squamous cell carcinoma cells (1, 9–12). Outside of cancer, FAP-α fibroblasts play a role in preserving tissue homeostasis in skeletal muscle, and FAP-α is expressed by PDGFR-α^+^, Sca-1^+^ multipotent bone marrow stromal cells (13). FAP-α is also implicated in human pathologies such as fibrosis, arthritis, atherosclerosis and autoimmune diseases (14).

Because of the expression of FAP-α by CAFs and by some malignant cells, there is heightened interest in targeting FAP-α in cancer. Downregulating FAP-α with siRNA in SKOV3 cells inhibited ovarian tumor growth in nude mice (15) whereas FAP-α overexpression in SKOV3 cells promoted ovarian cancer cell proliferation, drug resistance, invasiveness and migration *in vitro*, and tumor growth *in vivo* (BALB/c-nu/nu mice) (16). FAP-α and a catalytically inactive mutant of FAP-α in human breast cancer cells increased tumor growth *in vivo* and invasiveness in culture (17). In melanoma cells, however, transfection of FAP-α resulted in decreased tumorigenicity (18). Clearly, an expansion of the functional roles of FAP-α is necessary to expand our understanding of this important target in cancer and in other diseases.

More than 15-25% of all breast cancers are triple negative lacking expression of the estrogen receptor (ER), progesterone receptor (19), and the human epidermal growth factor receptor 2 (HER2) (20). These cancers tend to be more common in premenopausal women (21). Treatment for TNBC is challenging because TNBCs have low response to endocrine therapy or molecular targeted therapy, and are more aggressive (19–21).

Here, we engineered triple negative MDA-MB-231 human breast cancer cells with FAP-α overexpressed, to understand the role of FAP-α in cancer cell metabolism using ^1^H magnetic resonance spectroscopy (MRS). We also characterized the effect of FAP-α on proliferation, migration and invasion by these cells. We identified significant changes in metabolism that identify, for the first time, the role of FAP-α in cancer cell metabolism. We identified increased migration and invasion that are consistent with the known roles of FAP-α. We additionally determined the effects of FAP-α overexpression on the human fibrosarcoma HT1080 cell line. HT1080 cells overexpressing FAP-α displayed increased migration, accompanied by limited changes in metabolism, identifying the dependency of the metabolic changes on cell type.

## Materials and methods

### Cell culture

Triple negative MDA-MB-231 wild type (231 WT) human breast cancer cells and human fibrosarcoma HT1080 cells were obtained from American Type Culture Collection (ATCC, Manassas, VA, USA), and grown in DMEM medium supplemented with 10% fetal bovine serum (FBS; Sigma-Aldrich, St. Louis, MO). MDA-MB-231 and HT1080 cells stably expressing FAP-α (231-FAP and HT-FAP) were engineered by transducing these cells with lentivirus encoding the gene for human FAP (Accession No. NM_004460.3) that was subcloned into lentiviral vector pMA3211. Genetically engineered FAP-α overexpressing MDA-MB-231 cells were maintained in DMEM medium supplemented with 10% fetal bovine serum (FBS) and puromycin (4 µg/ml). 2 µg/ml puromycin was used for the HT-FAP cells. Cells were maintained in a humidified atmosphere with 5% CO2 in air at 37°C, and were tested routinely for mycoplasma contamination. Experiments were performed with puromycin free medium. Cell count and cell size measurements were performed using an automated cell counter (Invitrogen Countess, Thermo fisher scientific, Waltham, MA, USA).

### Cell viability/proliferation assay by CCK-8

Five thousand cells were seeded in each well of a 96 well plate and cultured overnight. At 24 h after cell seeding, cell viability was determined with a cell counting kit-8 assay (CCK-8, Dojindo Molecular Technologies, Inc. MD, USA), using the manufacturer’s instructions. Cells were cultured in fresh medium with or without 10% FBS and cell viability was determined at day 3 and day 4 using the CCK-8 assay. Cell viability was measured at 450 nm (A450) using a 1420 Multilabel counter (Perkin Elmer, Waltham, MA, USA) after 2 h incubation at 37°C with the CCK-8 reagent. In the CCK-8 colorimetric assay the amount of the formazan dye generated by the activity of dehydrogenases is directly proportional to the number of living cells. Cell viability was normalized to measurements at day 1. Doubling times of cells cultured in complete medium were determined using A450 values at day 1 (A1) and day 4 (A4) for 72 h culture (t) using the equation: doubling time = t*ln (2)/(ln(A4)-ln(A1)). Four independent experiments were performed.

### Immunoblot analysis

Immunoblots were obtained from cells cultured in medium with or without serum (1-3 days). Whole-cell extracts were prepared by lysing cells with RIPA lysis buffer supplemented with a protease inhibitor cocktail (Sigma-Aldrich, St Louis, MO, USA). Equal amounts of total protein (60 or 100 µg) were resolved on a 7.5% SDS-PAGE gels or 4-20% precast polyacrylamide gel (Bio-Rad Laboratories, Hercules, CA, USA) and transferred to a nitrocellulose membrane (Bio-Rad). After blocking in 5% milk-TBST (TBS Tween) or 5% BSA-TBST, the membrane was separately probed with antibodies against FAP-α (R&D Systems Inc AF3715. Minneapolis, MN, USA), choline kinase-alpha (Chk-α) (Proteintech Group, Chicago custom-made, IL, USA), tumor necrosis factor alpha (TNF-α), (Cell Signaling Technology #6945, Danvers, MA, USA), focal adhesion kinase (FAK) (Cell Signaling Technology #3285), and phosphorylated FAK (pFAK) (Thermo Fisher Scientific #700255). Anti-GAPDH antibody (Sigma-Aldrich G8795) was used for loading assessment. Secondary antibodies were horseradish peroxidase conjugated anti-sheep (R&D Systems Inc. F0128), anti-mouse (GE Healthcare NA931, Chicago, IL, USA), or anti-rabbit (GE Healthcare NA934). The signal was visualized using ECL Plus reagents (Thermo Fisher Scientific). Band intensities were measured using ImageJ and the relative intensities of FAP-α/GAPDH, Chk-α/GAPDH, FAK/GAPDH, pFAK/GAPDH, and TNF-α/GAPDH were obtained. Band intensity ratios for Chk-α, FAK, pFAK and TNF-α were normalized to the average values from 231 WT cells with FBS.

### Dual-phase extraction and ^1^H magnetic resonance spectroscopy analysis

Three million cells per flask were seeded, and cultured for 2 days in cultured media with FBS (+FBS), or medium was changed to serum free medium next day after seeding and cells were cultured for 2 more days (-FBS). Cells were collected by trypsinization and live cells were counted based on trypan blue exclusion. More than 20 million cells were used for cell extraction. Both water- and lipid- soluble extract fractions were obtained using a dual-phase extraction method. Briefly, pelleted cells were mixed with 3 mL of ice-cold methanol and gently vortexed. Six mL of chloroform were added and ultrasonication under ice-cold conditions was performed for 30 s 3 times with a 1s pulse interval. Finally, 2 mL of water were added and mixed with a sonicator. All procedures were performed on ice, and samples were stored at 4°C overnight for phase separation and later centrifuged at 7,500 *g* at 4°C for 20 min. The aqueous phase containing water-soluble metabolites was collected. Methanol in the aqueous phase was first evaporated under nitrogen gas, and any water remaining in the aqueous phase was lyophilized. Dried aqueous phase extracts were re-suspended in 0.6 mL of a buffer solution at pH 7.4 consisting of 89% deuterated water (D_2_O), 10% 10x PBS, 1% D_2_O with 0.75 wt % TSP (3-(trimethylsilyl) propionic 2,2,3,3-d4 acid) sodium salt used as an internal standard for ^1^H MRS. Lipid-soluble extracts were resuspended in 0.4 mL of chloroform-D and 0.2 mL of methanol-D4 with 0.05 v/v % tetramethylsilane (TMS) (Cambridge Isotope Laboratories, Inc., Tewksbury, MA, USA) used as an internal standard.

^1^H MR spectra of aqueous and lipid phase extracts were acquired on an Avance III 750 MHz MR spectrometer (Bruker BioSpin Corp. Billerica, MA, USA) equipped with a 5 mm probe. Spectra from the aqueous phase were acquired with water suppression using pre-saturation and a single pulse sequence with the following parameters: spectral width of 15495.87 Hz, data points of 64K, 90° flip angle, relaxation delay of 10 sec, acquisition time of 2.11 sec, 32 scans with 8 dummy scans, receiver gain 128. Spectra of lipid phase were acquired with the same parameters as aqueous phase without water suppression.

Spectra were analyzed using Bruker TopSpin 3.6.1 software (Bruker BioSpin Corp.). Metabolites were quantified as previously described (22). Integrals of resonances were determined and normalized to number of cells and compared to the TSP standard (aqueous phase) or TMS standard (lipid phase) to obtain relative concentrations in arbitrary units (A.U.).

### Wound healing assay

Two million 231 WT or 231-FAP cells per well, or one million HT1080 or HT-FAP cells were seeded on 6 well plates. To minimize proliferation effect, serum-free medium was added to each well the following day. After 24 h of serum starvation, cells were wounded with a p200 pipette tip, washed twice with HBSS and fresh serum free medium was added. Images from the wounded region were taken immediately (0 h), and at 6 h, 24 h and 48 h after wounding using an inverted microscope (Nikon Eclipse TS100) with a 4 x objective lens. Three to four images were analyzed using Fiji (ImageJ) at each time point to characterize changes in the wound area. Four individual experiments were performed.

### Invasion assay

Invasion assays were performed with a CultreCoat 96 well basement membrane extract (BME) cell invasion assay kit (Trevigen, Inc. Gaithersburg, MD) using the manufacturer’s instructions. Cells were starved overnight using serum-free medium and 2.5 x 10^4^ cells in serum-free medium were seeded in the top chamber. Medium with 10% FBS was added to the bottom chamber and the plate was incubated at 37°C in a CO_2_ incubator for 24 h following which cell invasion was quantified according to the manufacturer’s protocol. Invasion was characterized with cells from three different passages. Invasion was normalized to values obtained from 231 WT cells.

### Statistical analysis

Data are expressed as Mean ± Standard Error Mean (SEM). Statistical significance was evaluated using a one-tailed unpaired Student’s t-test. P values ≤ 0.05 were considered to be significant. Three or more samples were used for the data analysis, unless otherwise noted.

## Results

### Characterization of FAP-α protein and cell size in FAP-α overexpressing cells

**Figure 1:**
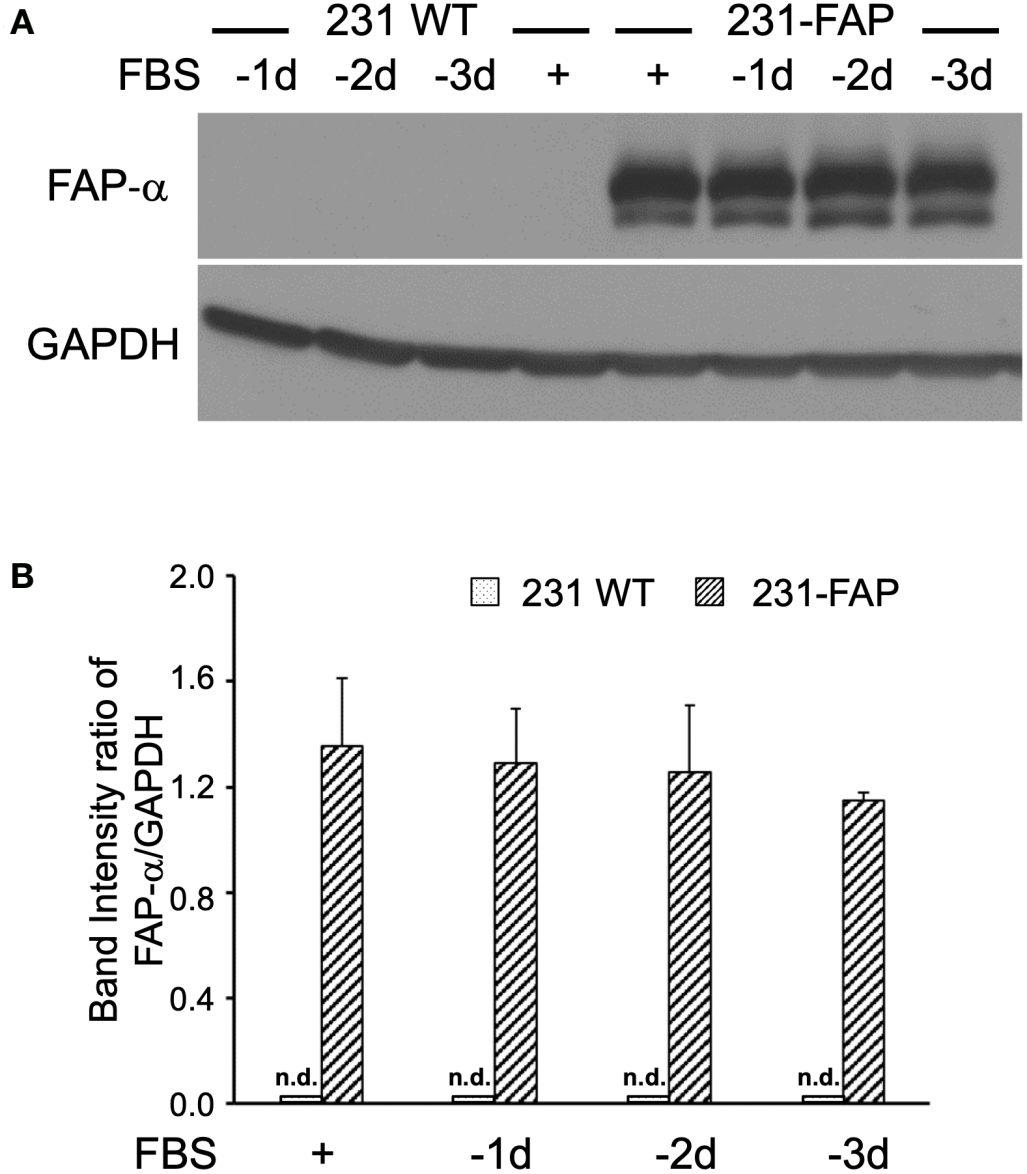
**(A)** Representative immunoblots of FAP-α with GAPDH used as loading control in 231 WT and 231-FAP cells cultured with (+) or without (–) FBS. -1d: 1 day without FBS. **(B)** Quantitative analysis of the immunoblots represented as band intensity ratio of FAP-α to GAPDH demonstrating significant increase of FAP-α in 231-FAP cells compared to 231 WT cells. Values represent Mean ± SEM (n = 2-3). n.d., non-detectable.

Expression levels of FAP-α protein were several fold higher in 231-FAP cells compared to the undetectable levels in 231 WT cells as shown in **Figures 1A, B**. FAP-α protein expression remained unchanged up to 3 days without serum. Similar results were obtained for HT-FAP cells as shown in **Supplementary Figures 1A, B**. Overexpression of FAP-α did not alter cell size in either cell line as determined from over 5,000 cells using an automated cell counter.

### Cell proliferation with CCK-8 assay

To investigate whether FAP-α altered proliferation either in the presence or absence of serum growth factors, cell proliferation rates were determined by a CCK-8 assay as shown in **Supplementary Figures 2A, B**. Cell proliferation of 231 WT and 231-FAP cells was not significantly different with or without serum, although 231-FAP had a slightly higher proliferation rate when supplemented with serum (**Supplementary Figure 2A**). Cell doubling times determined from CCK-8 data with serum at day 1 and day 4 were also comparable between 231 WT and 231-FAP cells with values of 30.34 ± 2.4 h *vs* 28.97 ± 2.2 h. Similarly, HT1080 WT and HT-FAP cells did not show a significant difference in doubling time with or without serum up to day 3 or without serum up to day 4 (**Supplementary Figure 2B**). However, HT-FAP cells showed a significant increase in doubling time with serum at day 4 compared to HT1080 WT cells.

### Cell metabolite levels with ^1^H MRS

**Figure 2:**
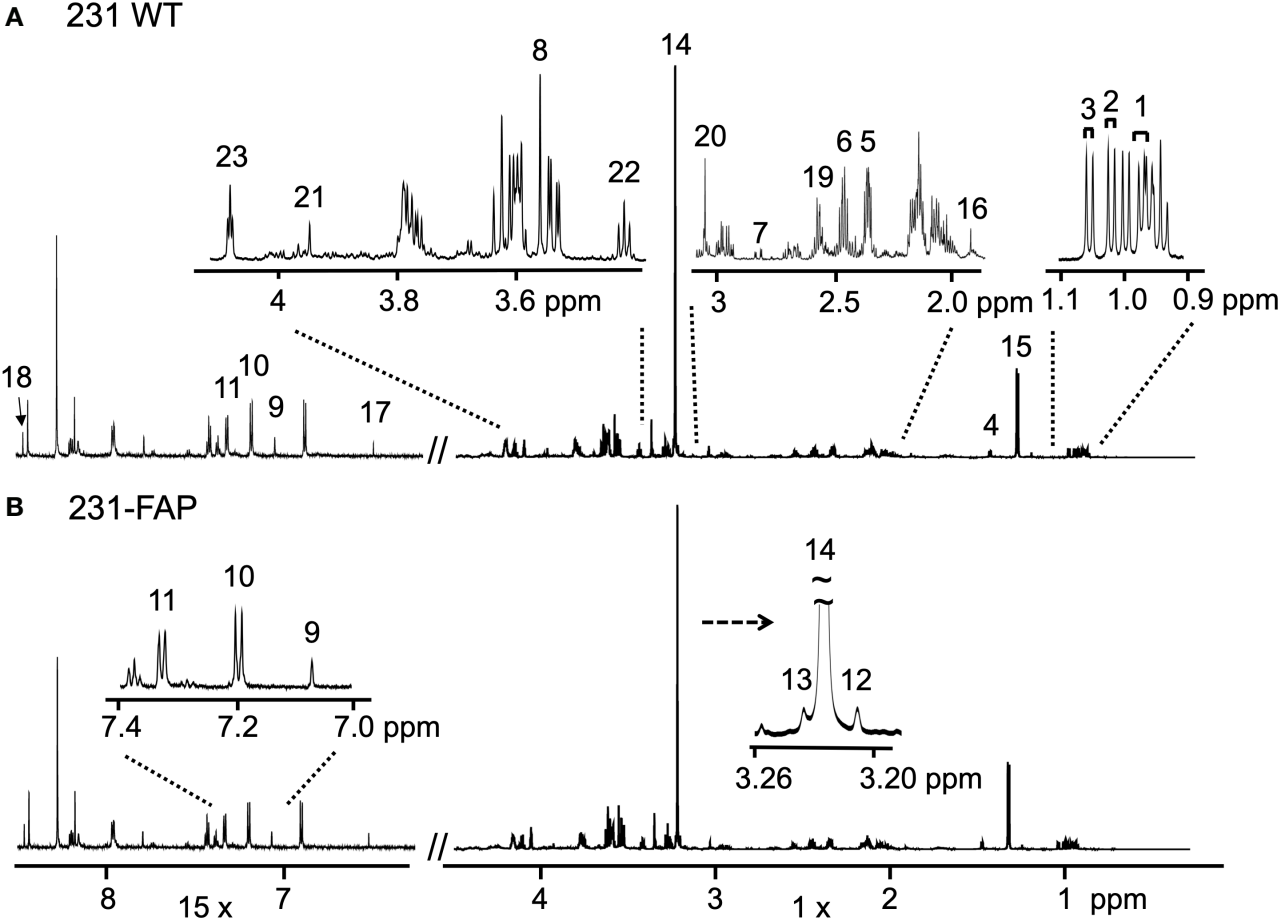
Representative ^1^H MR spectra obtained from the aqueous phase of **(A)** 231 WT and **(B)** 231-FAP cells cultured with serum. The spectral region from 6.3 - 8.5 ppm was magnified x15. Amino acids; 1: Leucine (Leu); 2: Isoleucine (Ile); 3: Valine (Val); 4: Alanine (Ala); 5: Glutamate (Glu); 6: Glutamine (Gln); 7: Aspartate (Asp); 8: Glycine (Gly); 9: Histidine (His); 10: Tyrosine (Tyr); 11: Phenylalanine (Phe). Choline metabolites; 12: Choline (Cho); 13: Glycerophosphocholine (GPC); 14: Phosphocholine (PC). Organic acids; 15: Lactate (Lac); 16: Acetate (Ace); 17: Fumarate (Fum); 18: Formate (For). Other metabolites; 19: Glutathione (GSH); 20: Creatine (Cr) and Phosphocreatine (PCr); 21: Cr; 22: Taurine (Tau); 23: Myo-inositol (MI).

To determine if FAP-α altered cell metabolism, we quantified water and lipid soluble metabolites obtained from ^1^H MR spectra of cell extracts. Cells were cultured with serum (+FBS) or without serum for 2 days (-FBS). Representative ^1^H MR spectra obtained from the aqueous phase of extracts from 231 WT and 231-FAP cells cultured with serum are presented in **Figures 2A, B**, respectively.

**Figure 3:**
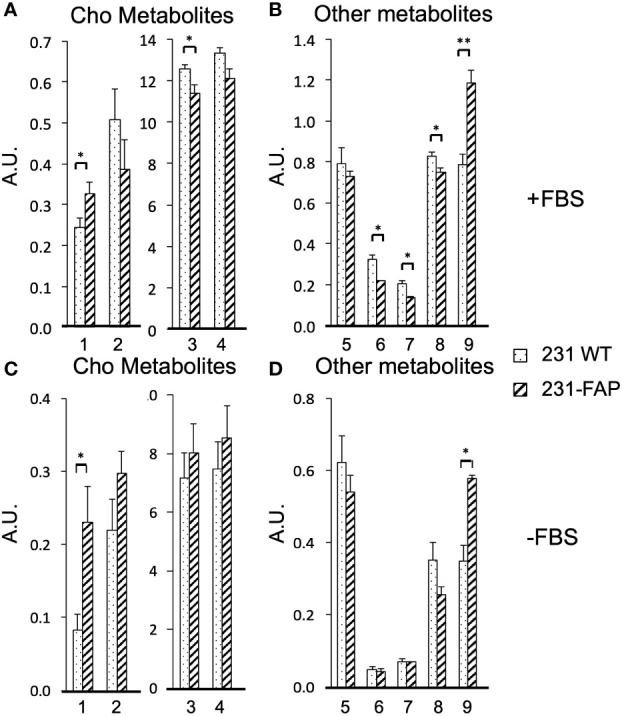
Relative concentrations in arbitrary units (A.U.) of **(A)** choline metabolites (1: Cho, 2: GPC, 3: PC, 4: total choline (tCho = Cho + PC + GPC) and **(B)** other metabolites (5: GSH, 6: Cr and PCr, 7: Cr, 8: Tau, 9: MI) from 231 WT and 231-FAP cells with FBS. **(C)** Choline metabolites and **(D)** other metabolites from 231 WT and 231-FAP cells without FBS (-FBS) for 2 d. ^1^H MRS data are from aqueous phase cell extracts. Values represent Mean ± SEM (n = 3). ** P < 0.01, * P ≤ 0.05.

A comparison of the relative metabolite concentrations in arbitrary unit (A.U.) in 231 WT and 231-FAP cells are presented in **Figures 3A** for choline metabolites and in **Figure 3B** for other metabolites for cells cultured with FBS (+FBS) and in **Figure 3C** for choline metabolites and **Figures 3D** for other metabolites for cells cultured without FBS (-FBS).

When 231 WT and 231-FAP cells were cultured with serum, significantly higher levels of choline (Cho) and myo-inositol (MI) and significantly lower levels of phosphocholine (PC), total creatine (Cr) (Cr + phosphoCr (PCr)), Cr alone and taurine (Tau) were observed in 231-FAP cells compared to 231 WT cells. The largest difference was observed in the increase of MI in 231-FAP cells. Withdrawing serum resulted in similar significant changes in Cho and MI.

There were no significant differences in amino acids (leucine, isoleucine, valine, alanine, glutamate, glutamine, aspartate, glycine, histidine, tyrosine, phenylalanine), and organic acids (lactate, acetate, fumarate, and formate) (**Supplementary Figure 3**).

FAP overexpression in HT1080 cells in serum free medium resulted in a significant increase of glycerophosphocholine (GPC) in serum free medium as shown in **Supplementary Figure 4**.

FAP overexpression did not alter MR detectable lipid metabolites including fatty acid, cholesterol, phosphatidylcholine + sphingomyelin, and phosphatidylethanolamine in either 231-FAP or HT-FAP cells as shown in **Supplementary Figures 5A–D**.

### Migration with wound healing assay

**Figure 4:**
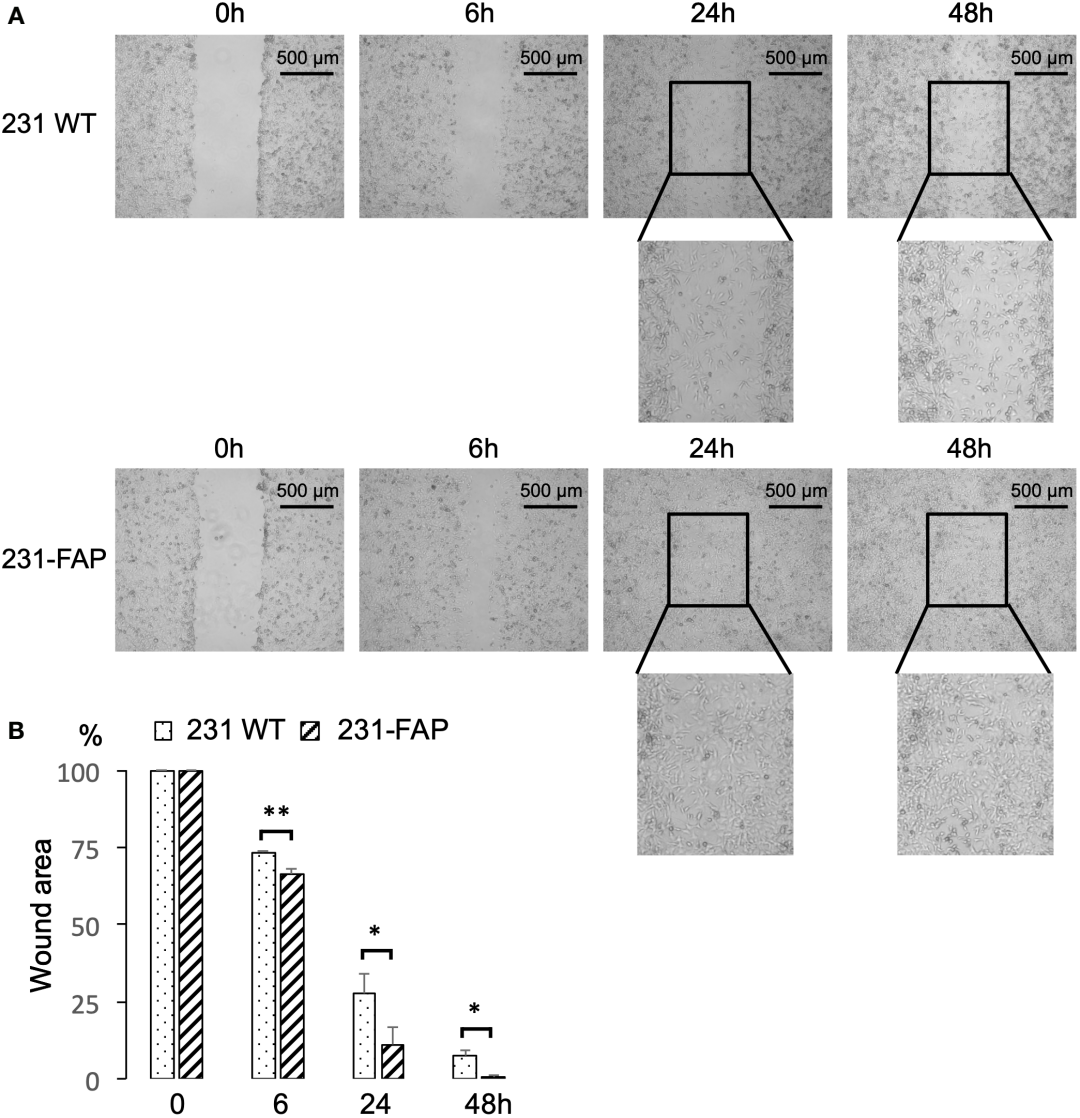
**(A)** Representative images from wound healing assay. Images were acquired at 0 h, 6 h, 24 h and 48 h after wounds were created. **(B)** Wound areas measured using ImageJ and compared to areas at 0 h (100%). Values represent Mean ± SEM (n = 4). ** P < 0.01, * P ≤ 0.05.

To investigate the effects of FAP-α on cell migration, we performed a wound healing assay. The assays were done with cells cultured in serum-free medium to avoid contribution from cell proliferation, as 231 WT and 231-FAP had comparable proliferation without serum. Representative images acquired at 0, 6, 24, and 48 h after cells were wounded are presented in **Figure 4A** to demonstrate the acceleration of wound closure by FAP cells compared to the WT cells. Almost complete closure of the wound by 48 h was observed with 231-FAP cells. Data averaged over four independent experiments are presented in **Figure 4B** confirming the significant reduction of the wound area in 231-FAP cells compared to 231 WT cells at all time points. By 48 h the wound area was almost undetectable in the 231-FAP cells.

We performed a wound healing assay with HT1080 cells to determine if we could replicate this observation in a different cell line. Similar to 231-FAP cells, wound closure by 48 h was observed in HT-FAP cells as shown in **Supplementary Figures 6A, B**. Like 231-FAP cells, under serum starvation there were no significant differences in proliferation between HT1080 WT and HT-FAP cells as previously shown in **Supplementary Figure 2B**.

### Invasion with basement membrane extract cell invasion assay

**Figure 5:**
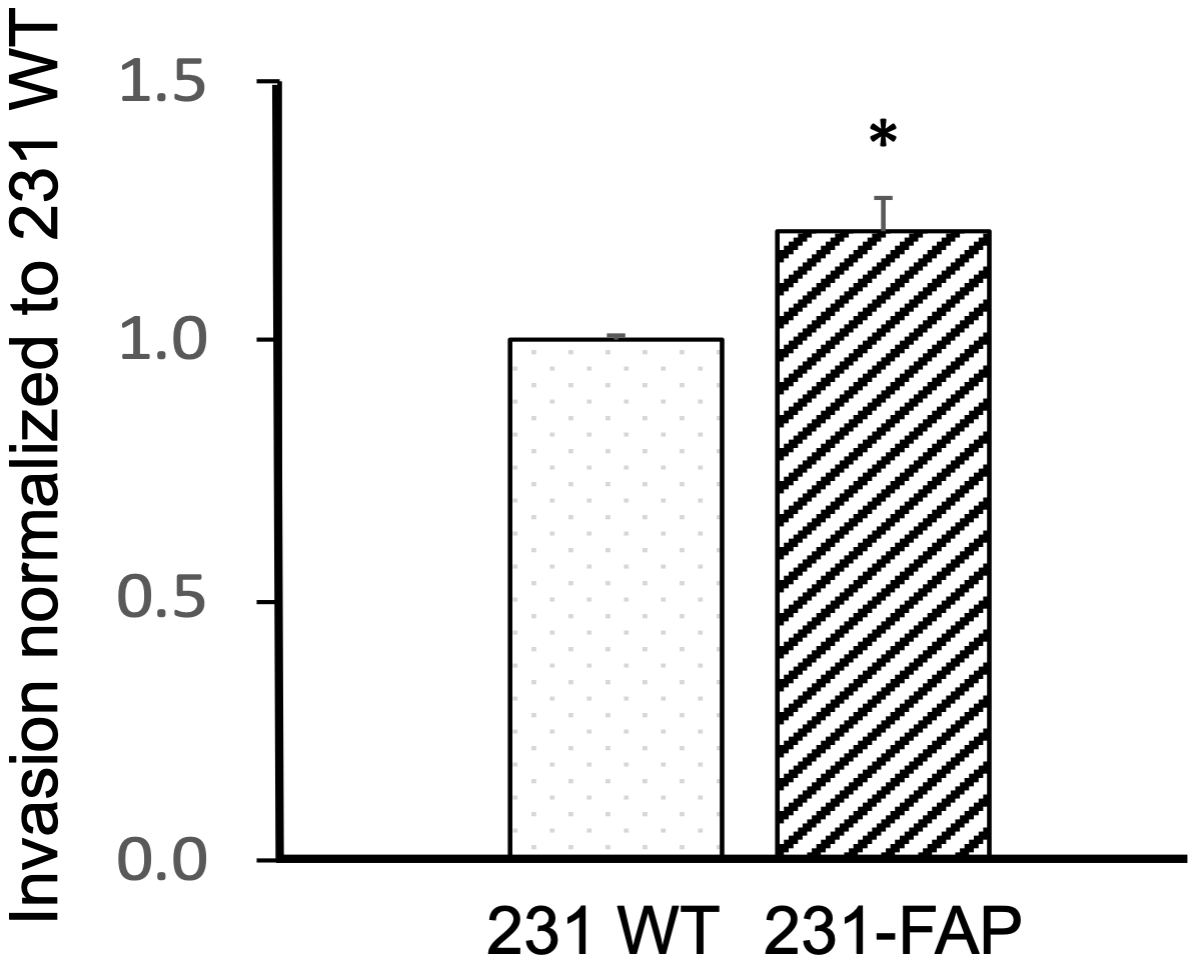
Invasion normalized to 231 WT cells in response to 10% FBS for 231 WT and 231-FAP cells (n = 3) over a 24 h period. Values represent Mean ± SEM. * P ≤ 0.05.

Since we observed significant differences in metabolism and migration with FAP expression in 231 cells, we further investigated the role of FAP-α in altering cell invasion, using a cell invasion assay. As shown in **Figure 5**, we detected a significant increase of ~21% invasion in 231-FAP cells compared to 231 WT cells.

### Protein levels with immunoblot analysis

**Figure 6:**
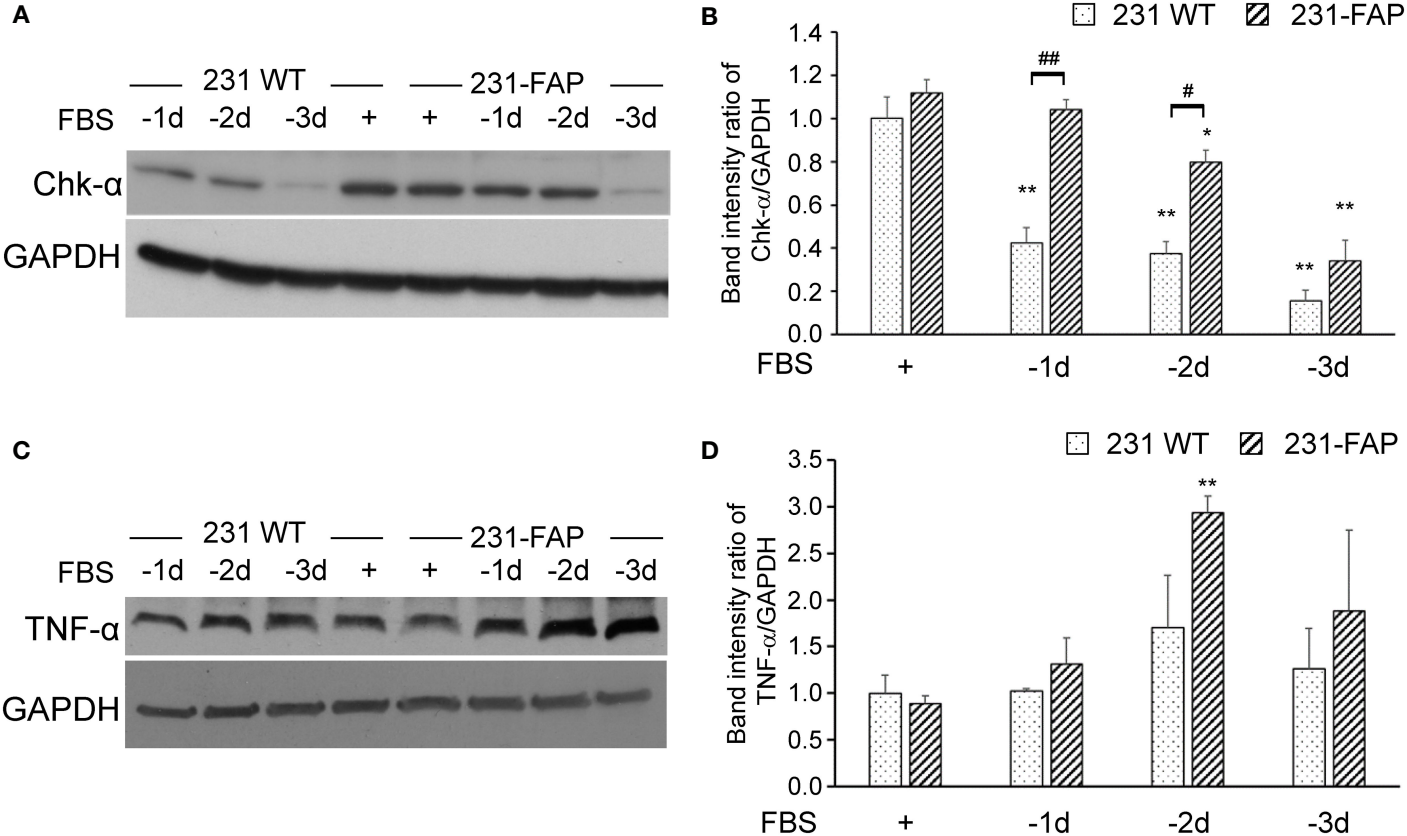
Representative immunoblots of **(A)** Chk-α and **(C)** TNF-α with GAPDH used as loading control in 231 WT and 231-FAP cells cultured with (+) or without (–) FBS. Quantitative analysis of immunoblots represented as band intensity ratio of **(B)** Chk-α (n = 2-3) and **(D)** TNF-α (n = 3) to GAPDH, normalized to 231 WT FBS+ values. -1d: 1 day without FBS. Values represent Mean ± SEM. ** P < 0.01, * P ≤ 0.05, for the same cell line. ## P < 0.01, # P ≤ 0.05 between 231 WT and 231-FAP cells.

Because we observed changes in choline metabolites with FAP-α overexpression, we characterized Chk-α protein levels using immunoblotting as shown in **Figures 6A, B**. Chk-α protein levels were comparable in 231 WT and 231-FAP cells when cultured in medium with 10% FBS. We further investigated Chk-α protein levels after serum starvation (1-3 days) since the wound healing and invasion assays were done in serum free medium. A reduction of Chk-α in 231 WT cells was observed within 1 day of serum starvation that progressively decreased with time, whereas 231-FAP cells maintained Chk-α levels up to 2 days after serum starvation. Chk-α level decreased in both cell lines by day 3 of serum starvation.

To further understand the molecular causes of increased wound healing and invasion, we characterized TNF-α in 231 WT and 231-FAP cells as shown in **Figures 6C, D**. Similar to the trend of Chk-α, TNF-α protein levels were comparable in 231 WT and 231-FAP cells when cultured with 10% FBS. Under serum starvation, however, TNF-α in 231-FAP showed a sustained increase starting from day 1 and over the 3 days of serum starvation compared to cells cultured with serum. By day 2 of starvation 231-FAP cells had significantly higher TNF-α compared to the same cells with FBS as shown in **Figure 6D**.

**Figure 7:**
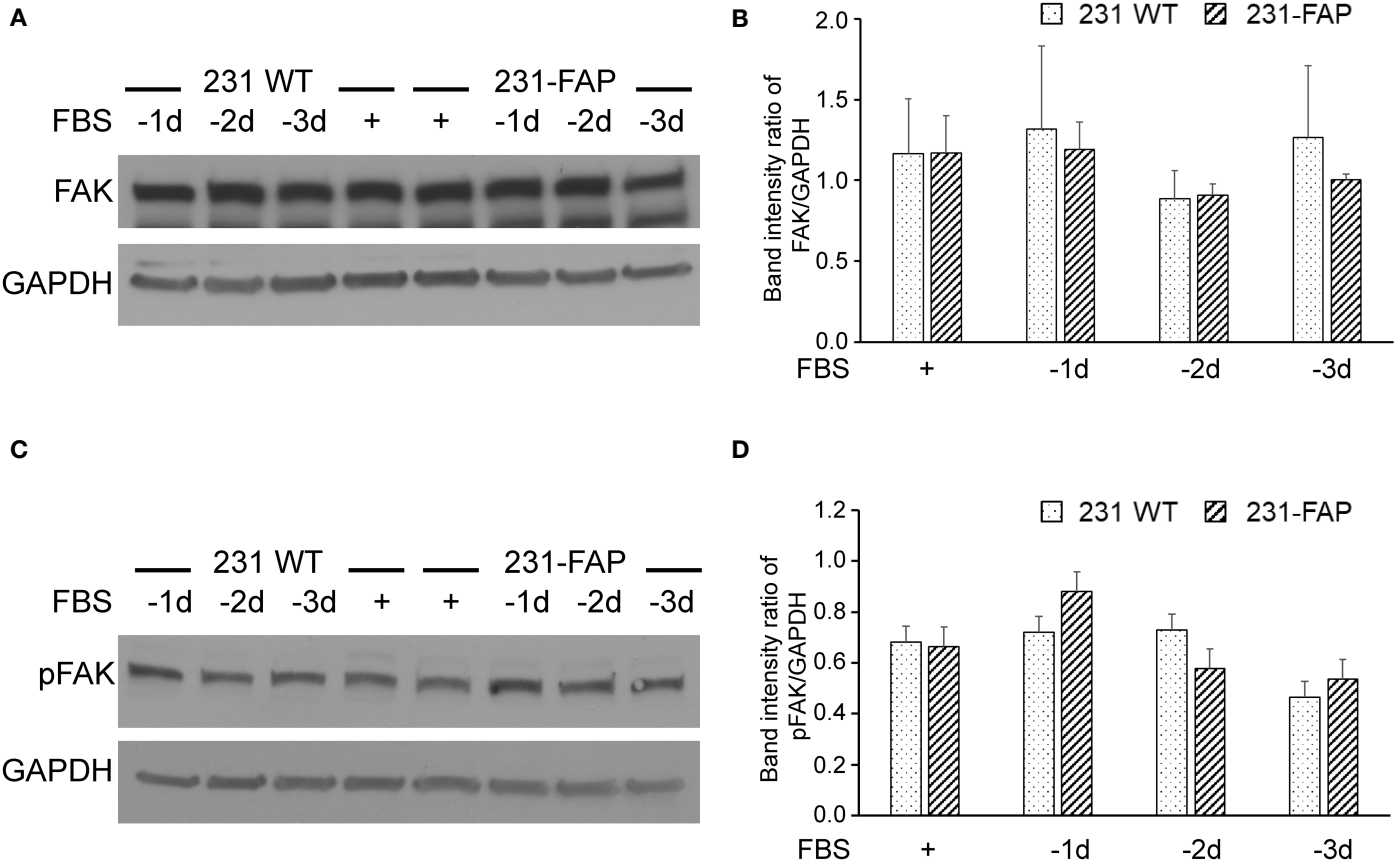
Representative immunoblots of **(A)** focal adhesion kinase (FAK) and **(C)** phosphorylated FAK (pFAK) with GAPDH used as loading control in 231 WT and 231-FAP cells cultured with (+) or without **(-)** FBS. Quantitative immunoblot analysis represented as band intensity ratio of **(B)** FAK (n = 2-3) and **(D)** pFAK (n = 3) to GAPDH, normalized to 231 WT FBS+ values. -1d: 1 day without FBS. Values represent Mean ± SEM.

We also investigated the effects of FAP-α on focal adhesion kinase expression (FAK). As shown in **Figure 7**, there were no differences in FAK (**Figures 7A, B**) and phosphorylated FAK (**Figures 7C, D**) expression levels between 231 WT and 231-FAP cells.

## Discussion

FAP-α is a transmembrane serine protease that is known to exhibit exopeptidase and endopeptidase/gelatinase/collagenase activity (1–4). Here we established, for the first time, that FAP-α overexpression in triple negative MDA-MB-231 breast cancer cells resulted in significant changes in metabolism. We observed acceleration of migration, as detected by a wound healing assay, and increased invasion. Cell proliferation, however, remained unaltered with FAP-α overexpression. FAP-α overexpression in the HT1080 fibrosarcoma cell line resulted in metabolic changes in GPC with a comparable effect in the wound healing assay, suggesting that metabolic changes induced by FAP-α may depend upon the cell type.

Of the metabolic changes observed in 231-FAP cells, the magnitude of the increase of myoinositol was the largest when cells were cultured with FBS. This significant change was consistent when cells were cultured without FBS for 2 days. Long known as a lipotropic factor, MI is a component of cell membrane phospholipids, a precursor of several second messengers such as inositol triphosphate (IP3), diacylglycerol (DAG), and inositolphosphoglycans (IPG) (23), and mediates osmoregulation (24). MI is an essential factor for cell survival and growth in normal and malignant human cells (25). In tumors, however, MI was found to have tumor suppressive effects (24). The anticancer effects of MI alone or in combination with inositol hexaphosphate (IP_6_) were shown in colon, breast, and metastatic lung cancer models (26–28). Further investigation is necessary to understand the FAP-α mediated increase of MI and its impact on the increased migration and invasion observed here.

Free choline also increased with FAP-α overexpression with and without FBS in MDA-MB-231 cells. Choline is converted to phosphocholine (PC) by Chk-α (29, 30). There were no differences in Chk-α protein levels when cells were cultured in medium containing 10% FBS. However, under serum starvation, Chk-α remained higher in 231-FAP cells compared to 231 WT cells. The potential role of FAP-α in increasing choline transport merits further investigation. Alterations of Cho and PC levels did not affect the level of PtdCho analyzed in the lipid phase of cell extracts.

With FBS, phosphocreatine, creatine, and taurine also significantly decreased with FAP-α overexpression suggesting changes in energy metabolism (31) and in osmoregulation that is mediated by taurine (32).

The absence of cell proliferation changes with FAP-α overexpression in 231-FAP cells are consistent with previous studies that showed that FAP-α overexpression in MDA-MB-231 cells increased tumor growth and vascularization without increasing cell proliferation (33, 34). Results from these studies suggest that FAP-α participates in “tumor-stroma cross-talk”, with the tumor microenvironment playing an important role in FAP-α promoting tumor growth.

The accelerated wound closure and migration observed by us is different from previous studies (33) where FAP-α overexpression in MDA-MB-231 cells decreased migration by reducing the level of phosphorylated focal adhesion kinase (pFAK) level which is reported as a modulator of migration and invasion (35). These previous studies were performed over a relatively short time-window of 4 h and without serum starvation. Although we did not observe a decrease of FAK or pFAK with or without starvation, our wound healing migration assay was performed with serum free medium to minimize effects due to cell proliferation.

Similar to MDA-MB-231 cells, HT1080 cells also showed accelerated wound healing with FAP-α overexpression. The increase of migration and invasion observed here are consistent with the known roles of FAP-α as an exopeptidase and endopeptidase/gelatinase/collagenase in tissue remodeling and repair, and in cell migration (1–4). FAP-α overexpressing fibroblasts were found to promote pancreatic cancer cell invasion by remodeling the stromal extracellular matrix (36). The increased migration and invasion is also supported by our observations that unlike 231 WT cells that showed a decrease, 231-FAP cells maintained Chk-α levels higher than 231 WT cells or up to 2 days of serum starvation. TNF-α showed a sustained increase starting from day 1 and over the 3 days of serum starvation in 231-FAP cells compared to 231 WT cells. Chk-α promotes an aggressive phenotype (37–41). Exogenous TNF-α has been shown to promote breast cancer cell migration accompanied by an increased secretion of MMP9, as well as upregulate the expression of CD26 and FAP-α in a dose-dependent manner (42).

Our studies have identified new functional roles of FAP-α that expand our understanding of this important target in triple negative MDA-MB-231 human breast cancer cells. The metabolic changes observed in MDA-MB-231 cells were not replicated in HT1080 cells suggesting that the role of FAP-α in altering metabolism may depend upon the cell type. Future studies should investigate changes induced by FAP-α overexpression in CAFs as well as in other cancer cells. These studies should also expand upon the molecular mechanisms underlying the role of FAP-α in inducing metabolic changes.

## Data availability statement

The raw data supporting the conclusions of this article will be made available by the authors, without undue reservation.

## Author contributions

Conceptualization: NM, BK, JJ and ZB. Methodology, NM, BK, YM, FW, JJ, FV, JB. Validation, NM, BK, JJ, YM, FW. Formal analysis, NM, FV, ZB. Writing-original draft preparation, NM. Writing-review and editing, NM, ZB. Project administration, ZB. Funding acquisition, ZB. All authors contributed to the article and approved the submitted version.
