# Supplementary material for: Functional roles of FAP-α in metabolism, migration and invasion of human cancer cells
Source: Front Oncol. 2023 Mar 1;13:1068405. doi: 10.3389/fonc.2023.1068405 (PMC10015381; doi:10.3389/fonc.2023.1068405)

Supplemental Figure 1: (A) Representative immunoblots of FAP-α with GAPDH used as loading control in HT1080 WT and HT-FAP cells cultured with (+) without (-) FBS. -1d: 1 day without FBS. **(B)** Quantitative analysis of the immunoblots represented as band intensity ratio of FAP-α to GAPDH demonstrating significant increase of FAP-α in HT-FAP cells compared to HT1080 WT cells. Values represent Mean ± SEM (n = 3). n.d.: non-detectable.

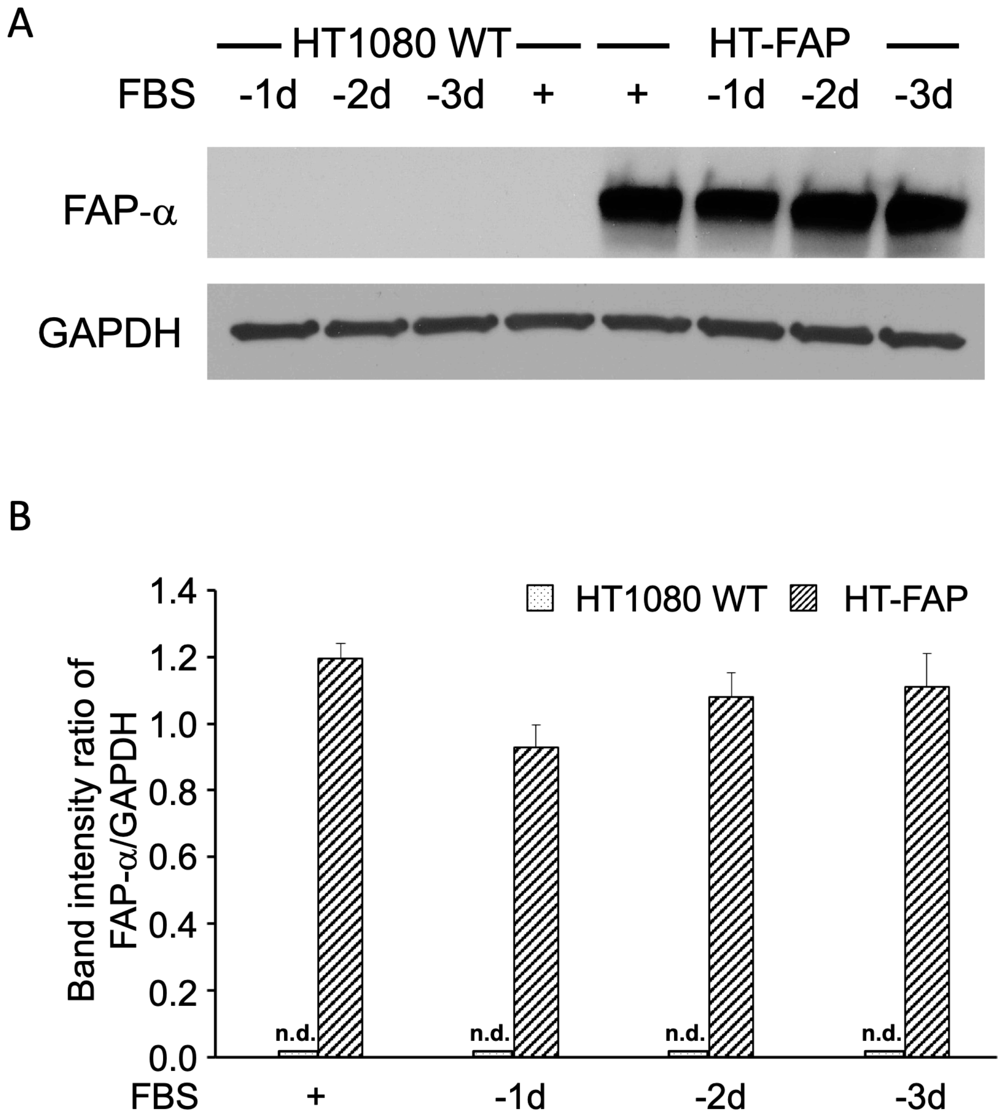

Supplemental Figure 2: Cell numbers obtained with the CCK-8 assay normalized to Day 1 of **(A)** 231 WT and 231-FAP cells (n = 4) and **(B)** HT1080 WT and HT-FAP cells (n=3-4). Values represent Mean ± SEM. ** P < 0.01

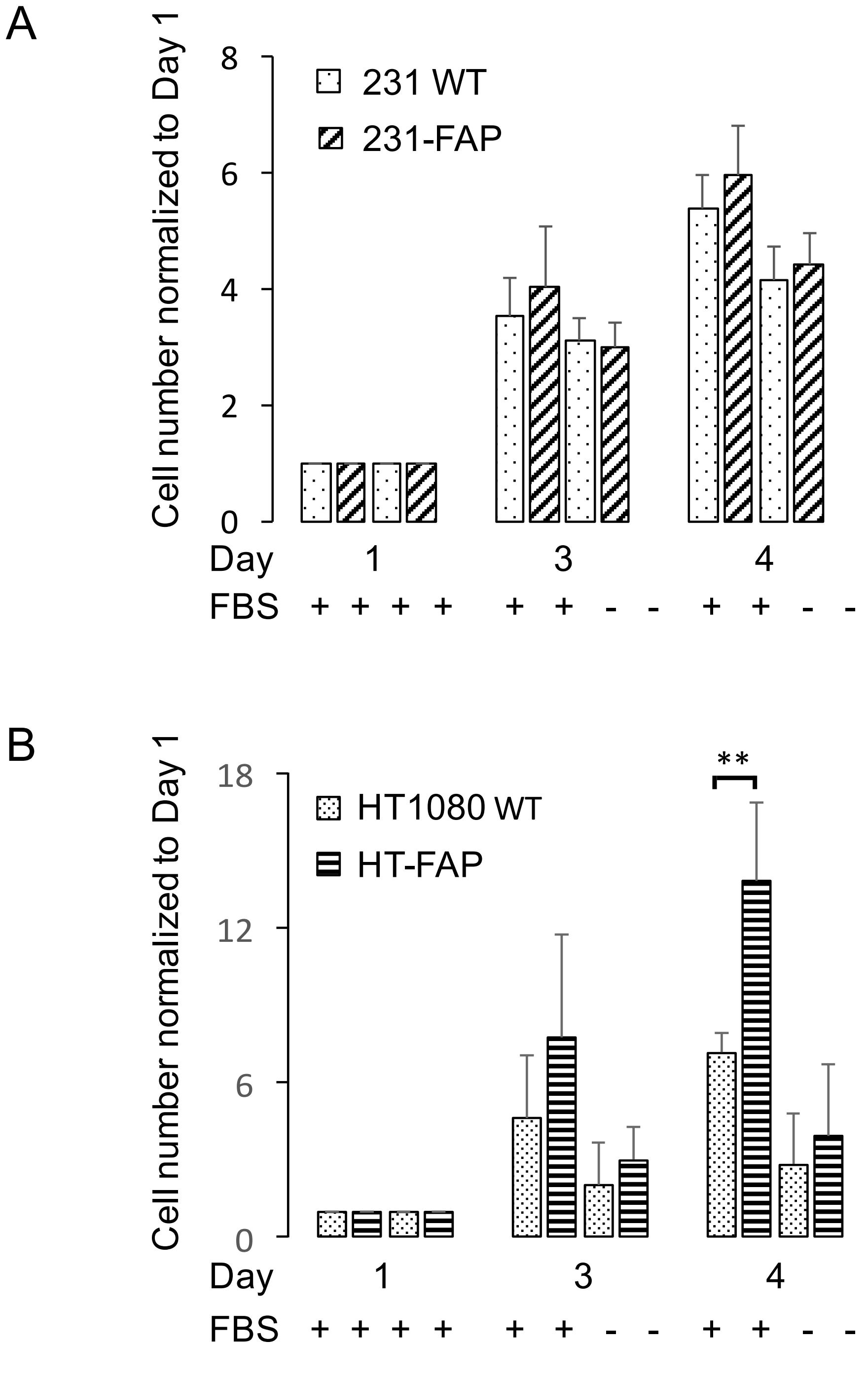

Supplemental Figure 3: Relative concentrations in arbitrary units (A.U.) of amino acids (1: Leu, 2: Ile, 3: Val, 4: Ala, 5: Glu, 6: Gln, 7: Asp, 8: Gly, 9: His, 10: Tyr, 11: Phe) from **(A)** 231 WT and 231-FAP cells with FBS (+FBS) and **(C)** without FBS (-FBS). Relative concentrations in arbitrary units (A.U.) of organic acids (12: Lac, 13: Ace, 14: Fum, 15: For) for 231 WT and 231-FAP cells **(B)** with FBS (+FBS) and **(D)** without FBS (-FBS). Amino acids and organic acids were obtained from ^{1}H MR spectra of aqueous phase cell extracts. Values represent Mean ± SEM (n = 3).

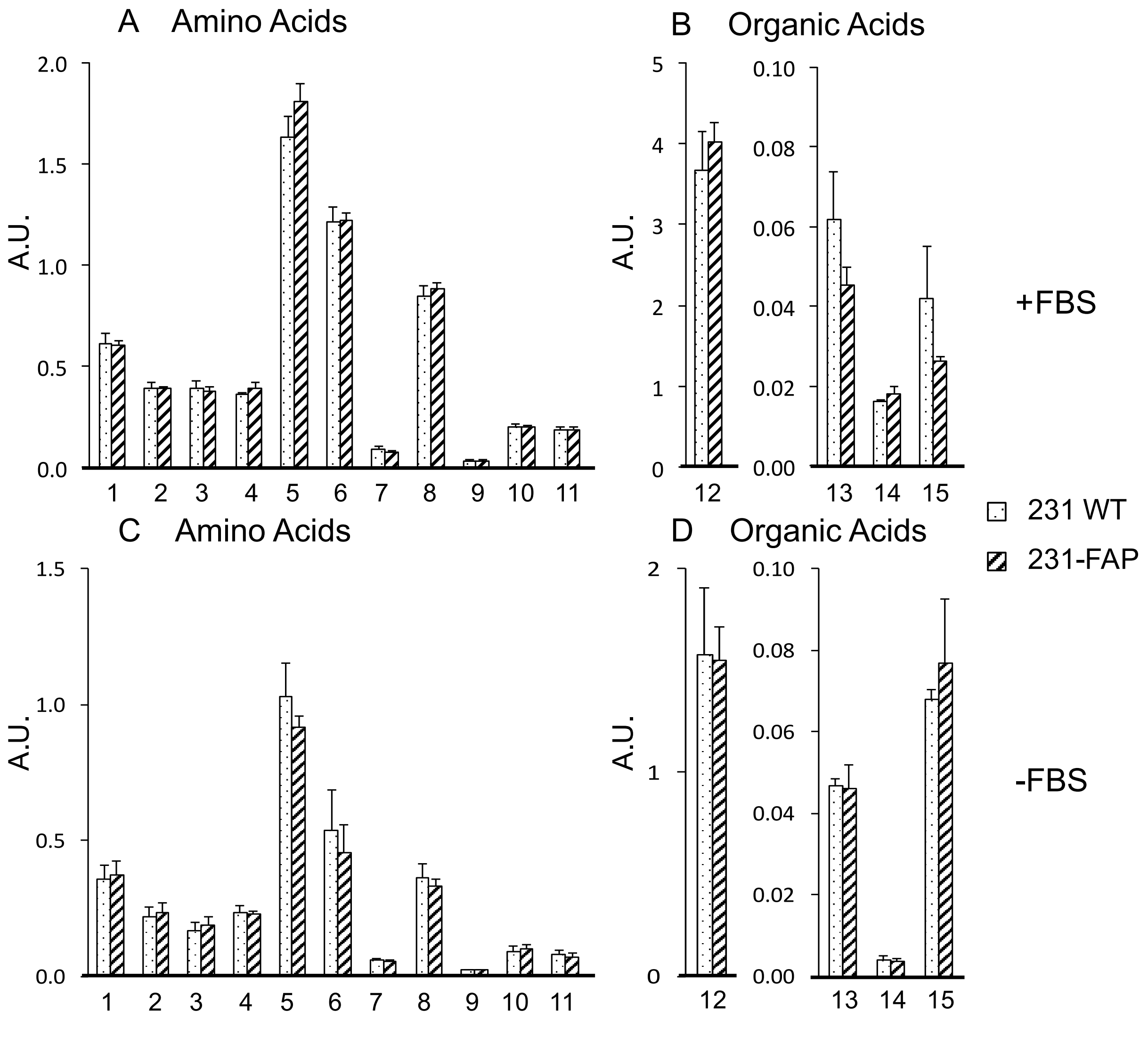

Supplemental Figure 4: Relative concentrations in arbitrary units (A.U.) of **(A and E)** amino acids (1: Leu, 2: Ile, 3: Val, 4: Ala, 5: Glu, 6: Gln, 7: Asp, 8: Gly, 9: His, 10: Tyr, 11: Phe), **(B and F)** organic acids (12: Lac 13: Ace, 14: Fum, 15: For), **(C and G)** choline metabolites (16: Cho, 17: GPC, 18: PC, 19: total choline (tCho = Cho + PC + GPC)), and **(D and H)** other metabolites (20: GSH, 21: Cr and PCr, 22: Cr, 23: Tau, 24: MI) in HT1080 WT and HT-FAP cells cultured with FBS **(A-D)** and without FBS for 2 days **(E-H)**. ^{1}H MRS data are from aqueous phase cell extracts. Values represent Mean ± SEM (n = 3). * P ≤ 0.05, between HT1080 WT and HT-FAP cells.

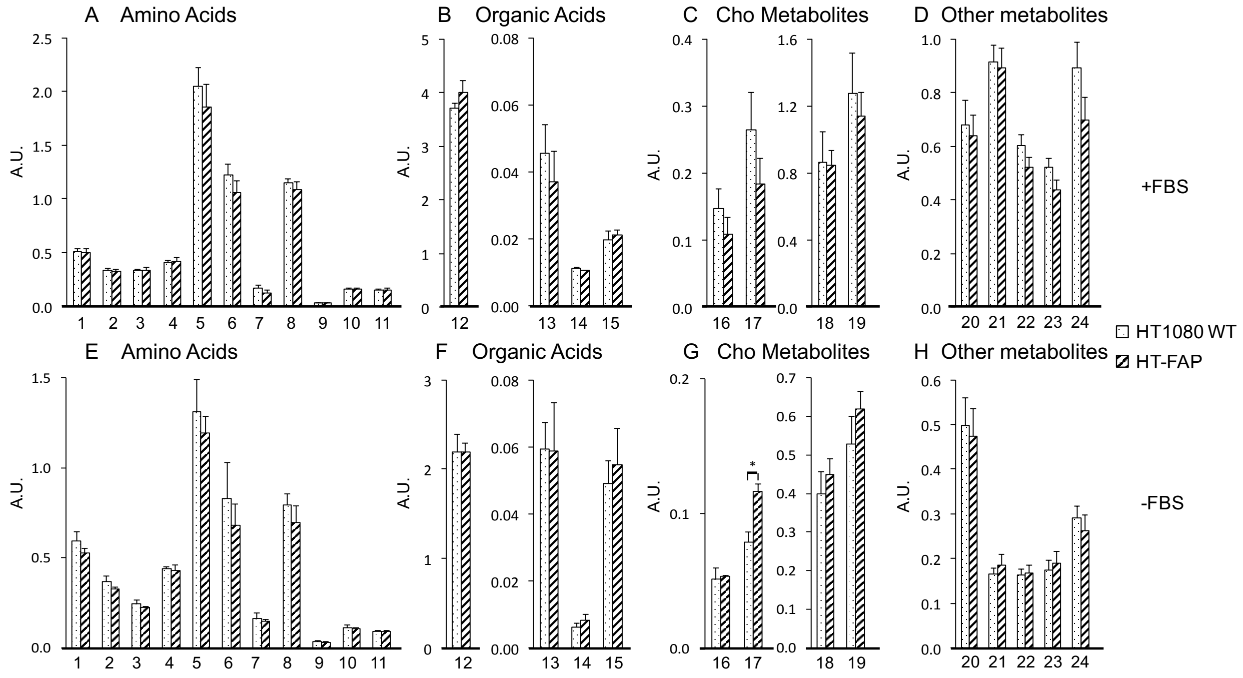

Supplemental Figure 5: Relative concentrations in arbitrary units (A.U.) of lipid metabolites obtained from ^{1}H MR spectra of lipid phase cell extracts (1: Fatty acids (-CH_3), 2: Fatty acids (-CH_2)n, 3: Cholesterol (-C18), 4: phosphatidylethanolamine, 5: -$N(CH_3)_3$ including phosphatidylcholine and sphingomyelin in **(A)** 231 WT and 231-FAP cells, and in **(B)** HT1080 WT and HT-FAP cells with FBS (+FBS). The same lipid metabolites in **(C)** 231 WT and 231-FAP cells, and in **(D)** HT1080 WT and HT-FAP cells without FBS (-FBS). Values represent Mean ± SEM (n = 3).

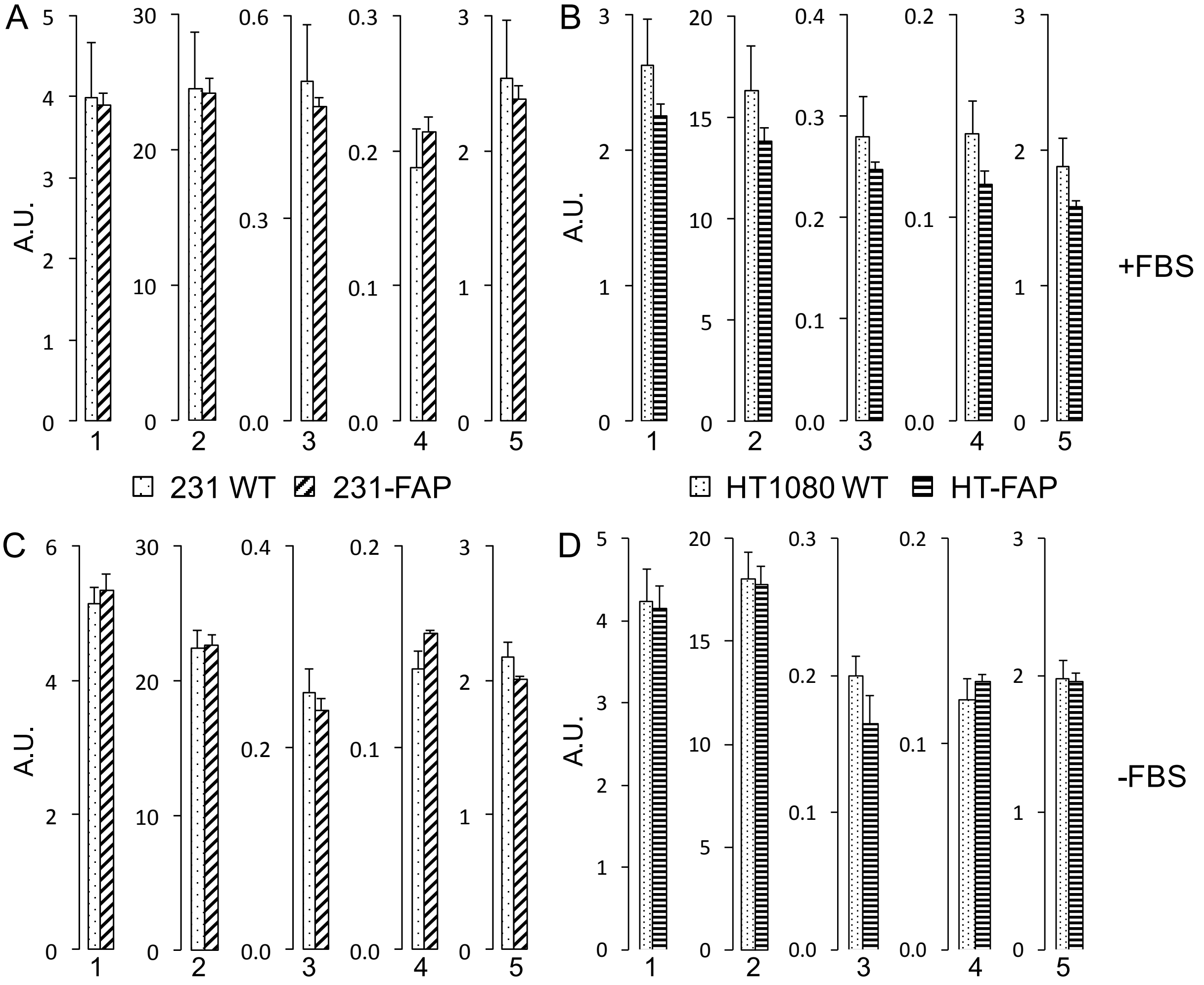

Supplemental Figure 6: (A) Representative images from wound healing assay. Images were acquired at 0 h, 6 h, 24 h and 48 h after wounds were created. **(B)** Wound areas measured using ImageJ and compared to areas at 0 h (100%). Values represent Mean ± SEM (n = 7). * P ≤ 0.05 between HT1080 WT and HT-FAP cells.

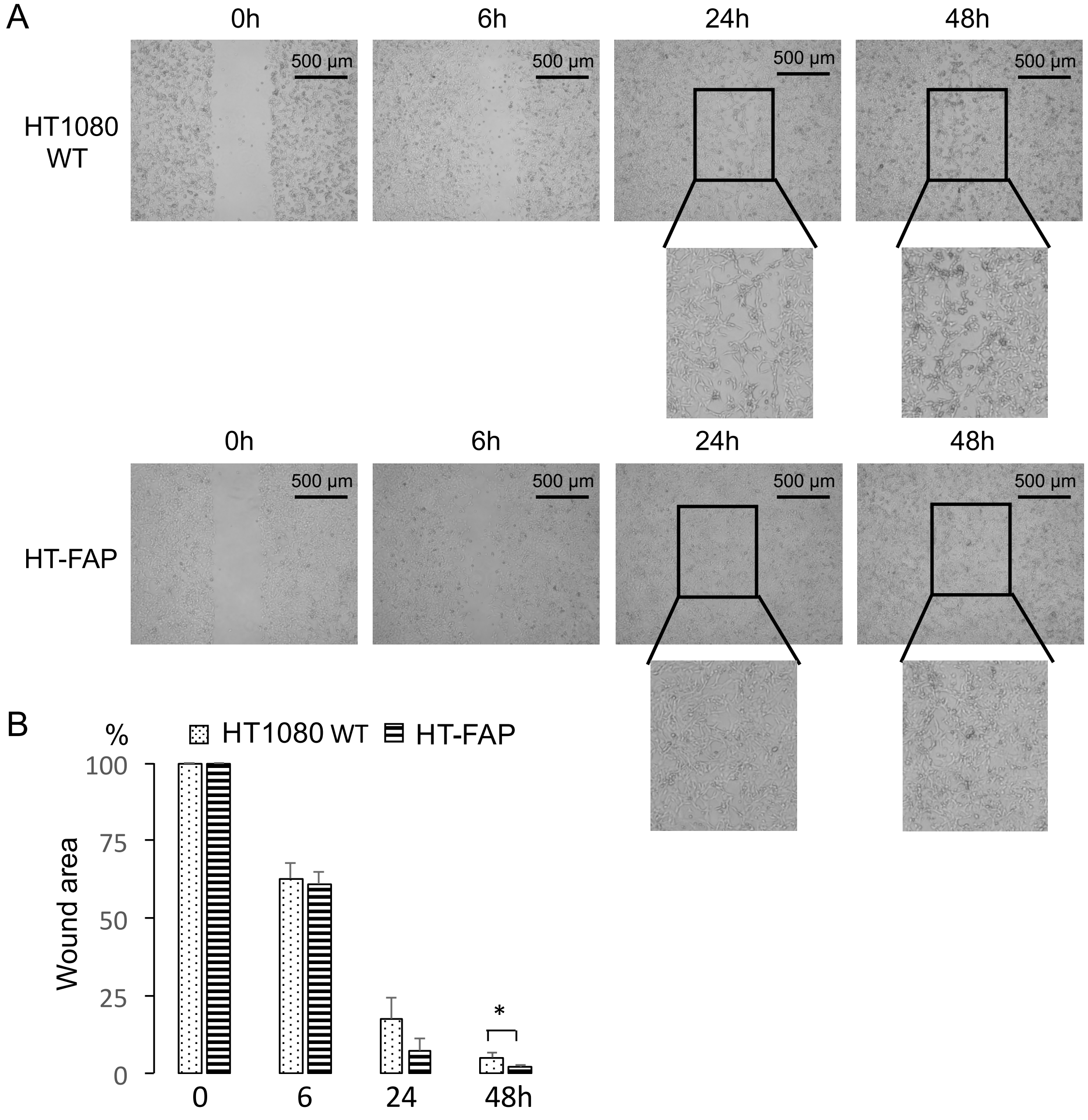

Supplement: Supplementary file 1 [file DataSheet_1.pdf]
